# National Delphi consensus on laser and energy‐based treatments for rosacea

**DOI:** 10.1111/ddg.15976

**Published:** 2025-12-19

**Authors:** Lynhda Nguyen, Nikolaus Seeber, Uwe Paasch, Martin Schaller, Claudia Borelli, Syrus Karsai, Gerd Kautz, Tanja C. Fischer, Michael Drosner, Stefan Hammes, Konstantin Feise, Christian Raulin, Martina Kerscher, Gerd Gauglitz, Jens M. Baron, Stefan W. Schneider, Katharina Herberger

**Affiliations:** ^1^ Department of Laser and Aesthetic Department of Dermatology and Venereology University Medical‐Center Hamburg‐Eppendorf Hamburg Germany; ^2^ Joint Practice for Dermatology Dres. Peter/Seeber/Altheide Hamburg Germany; ^3^ Department of Dermatology Venereology and Allergy University of Leipzig Leipzig Germany; ^4^ Department of Dermatology University Hospital Tübingen Tübingen Germany; ^5^ Aesthetic and Laser Unit Department of Dermatology Eberhard Karls University Tuebingen Tuebingen Germany; ^6^ Dermatologikum Hamburg Hamburg Germany; ^7^ Skin and Laser Clinic Dr. Kautz Konz Germany; ^8^ Skin and Laser Center Potsdam Germany; ^9^ Skin & Laser Schwerin Schwerin Germany; ^10^ Department for Oral and Maxillofacial Surgery/Plastic Surgery University of Greifswald Greifswald Germany; ^11^ Sophienklinik Stuttgart Germany; ^12^ Laserklinik Karlsruhe GmbH Karlsruhe Germany; ^13^ Division of Cosmetic Sciences Department of Chemistry University of Hamburg Hamburg Germany; ^14^ Haut‐ und Laserzentrum Glockenbach Munich Germany; ^15^ Department of Dermatology and Allergology University Hospital RWTH Aachen Aachen Germany; ^16^ Department of Dermatology and Venereology University Medical‐Center Hamburg‐Eppendorf Hamburg Germany

**Keywords:** Delphi consensus, energy‐based systems, lasers, non‐ionizing beam sources, rosacea

## Abstract

**Background:**

Various treatments are available for rosacea, including lasers and energy‐based devices (EBDs). However, the specific role of lasers and EBDs in rosacea management remains insufficiently defined in Germany. The aim of the study was to develop a comprehensive expert consensus on the use of lasers and EBDs in the treatment of rosacea.

**Patients and Methods:**

A three‐phase study was conducted. First, a steering group performed a systematic literature review and meta‐analysis. Second, a prospective, multicenter online survey gathered patient‐reported experiences and treatment perspectives. Third, a structured, three‐round Delphi process was implemented to discuss consensus statements based on the literature and patient input.

**Results:**

A total of 185 patients participated in the survey. Based on the systematic review and patient questionnaire consensus was performed on *(1)* the role of lasers and EBDs across different rosacea phenotypes, *(2)* the integration of laser/EBD treatments with combination therapies, and *(3)* best practices for pre‐ and post‐treatment care. 16 dermatologists contributed to the Delphi process.

**Conclusions:**

This expert consensus offers practical guidance for incorporating laser and EBD therapies into rosacea treatment strategies. The Delphi process clarifies key aspects of their role in management, their integration into combination therapies and directions for future research.

## INTRODUCTION

Rosacea is a prevalent chronic inflammatory skin disorder that primarily affects the centrofacial region, including the cheeks, nose, forehead, and chin. It is characterized by a variable clinical presentation, often beginning with flushing that progresses to persistent erythema and telangiectasia. As the disease advances, inflammatory lesions such as papules and pustules may develop, and in some cases, phymatous changes can occur.^1^ In addition to visible skin manifestations, patients frequently report sensory symptoms such as burning, stinging, dryness, and itching, further contributing to discomfort and disease burden. Beyond its physical symptoms, rosacea has a profound impact on patients' psychological well‐being and overall quality of life. Studies have demonstrated that individuals with rosacea often experience emotional distress, social anxiety, and depression due to the chronic nature of the disease.^2^, ^3^

A wide range of therapeutic options are available for rosacea, including topical and systemic pharmacologic treatments, laser and energy‐based devices (EBDs), tailored dermocosmetic regimens, and lifestyle modifications. The most recent German guideline on rosacea (2022) provides a comprehensive overview of these approaches.^4^ However, despite growing evidence supporting the use of laser and EBDs in rosacea treatment, their precise role within the treatment algorithm remains incompletely defined.^5^, ^6^, ^7^, ^8^ While pharmacologic treatments are often effective in controlling inflammatory lesions, they may not adequately address persistent erythema and telangiectasia, which are among the most distressing symptoms for patients.^9^

Lasers and EBDs offer a targeted, non‐invasive approach to manage vascular and inflammatory components of rosacea. Various modalities, including pulsed dye laser (PDL), intense pulsed light (IPL), and Nd:YAG laser, have demonstrated efficacy in reducing erythema, vascular lesions, and overall disease severity.^10^ However, due to variations in disease presentation, treatment protocols, device parameters, and patient selection criteria, there is no standard of care for their use in rosacea. This lack of consensus creates uncertainty among both clinicians and patients regarding the role of these technologies.

Given the discrepancy between emerging clinical evidence and existing guideline recommendations, there is a pressing need for a standardized, evidence‐based approach to integrate lasers and EBD therapies into rosacea management. The present consensus work is based on a three‐stage development process consisting of a meta‐analysis, a national patient survey, and a multi‐stage Delphi expert consensus. The aim was to establish a comprehensive consensus on the role of lasers and EBDs in the treatment of rosacea. By addressing current knowledge gaps and defining best practices, this study seeks to optimize treatment strategies, improve patient outcomes, and provide clinicians with guidance on the effective use of these advanced therapeutic modalities.

## MATERIALS AND METHODS

### Study Design

The study adhered to the guidelines outlined in the *Accurate Consensus Reporting Document* (ACCORD). Further details can be found in the online supplementary Material S1. The research was structured as a three‐phase study. First, a steering group performed a comprehensive literature review and meta‐analysis. Second, a prospective, multicenter survey was carried out using an online questionnaire to capture rosacea patients' experiences and perspectives. Third, a structured three‐round electronic Delphi process was conducted to refine and validate consensus statements derived from the literature and patient feedback. It was performed using an institution‐based software platform (LimeSurvey V6.1.8, Hamburg, Germany) and took place between May 2023 and August 2024.

### Steering committee and literature search

Before formulating the consensus statements, a systematic literature search was conducted using *Medline*, *Web of Science*, and the *Cochrane Central Register of Controlled Trials* (CENTRAL). Following keywords were used:

#1 rosacea AND laser*
#2 rosacea AND photodynamic therapy
#3 rosacea AND flash lamp
#4 rosacea AND intense pulsed light
#5 rosacea AND radiofrequency

Additionally, forward and backward citation searches were performed to identify further, relevant studies. Ongoing clinical trials were searched in the World Health Organization Trial Registry and ClinicalTrials.gov. The articles retrieved were systematically reviewed and contributed to publish a network meta‐analysis evaluating the efficacy and safety of different laser‐ and light‐based treatments for rosacea.^11^

### Patient Questionnaire

To incorporate patient perspectives alongside scientific expertise, each participating expert was encouraged to invite their rosacea patients to contribute anonymously through an online questionnaire. The questionnaire is provided in online supplementary Material S2. Additionally, the national patient advocacy group “Deutsche Rosazea Hilfe e.V.” was invited to include its members in the survey.

### Panel Selection

Board‐certified dermatologists in Germany with at least one first or senior authorship in the field or involvement in relevant medical guidelines were invited to participate in the consensus panel. To ensure diversity and broaden the panel's composition, additional experts were subsequently invited. Members of the steering group did not function as panelists.

### Modified Delphi consensus

A modified Delphi consensus methodology was applied. Based on the literature review and patient questionnaire, the steering committee identified key areas requiring national consensus: *(1)* the role of lasers and EBDs in treating different rosacea phenotypes, *(2)* the use of combination therapies incorporating laser and EBD treatments, *(3)* best practices for pre‐ and post‐treatment care in laser and EBD procedures. The steering group initially formulated a set of preselected statements with multiple‐choice response options. Panelists were given the opportunity to provide additional comments or suggest modifications via an optional free‐text field. All statements were sent to the expert panel via email, with integrated reminder notifications. Panelists were asked to indicate whether they agreed, disagreed, or abstained from responding to each statement. Anonymity was maintained throughout the process to ensure that panelists remained blinded to each other's identities and individual responses.

### Statistical analysis

Statistical analyses were conducted using SPSS Statistics (IBM, Version 29.0.2.0). Descriptive statistics were used to summarize participant demographics. Mean values, standard deviations and ranges (minimum–maximum) were reported. A p value of < 0.05 was considered statistically significant. Agreement levels for each Delphi statement were expressed as percentages. Statements achieving ≥ 75% agreement or disagreement were considered to have reached consensus and were not included in subsequent rounds. Statements that did not meet consensus were revised based on panelist feedback and re‐evaluated in the next round.

## RESULTS

### Patient questionnaire

#### Basic Characteristics

A total of 185 patients participated in the online‐based questionnaire. The average age of the respondents was 53.5 ± 11.7 years (27–81), with the majority being female (78.7 %). On average, patients underwent 5.1 ± 5.5 laser treatment sessions (1–40). Among the participants, 42.5 % reported having completed their laser treatment with 3.3 ± 2.8 sessions (1–12).

#### Psychosocial impact

The psychosocial impact of rosacea was assessed using a six‐point scale, ranging from 1 (none) to 6 (severe). The negative effect of rosacea on daily life was rated at an average of 3.9 ± 1.5 (1–6), while the perception of stigmatization was scored at 2.4 ± 1.5 (1–6). Many patients (64.4 %) reported experiencing restrictions in their daily lives due to rosacea, with the most frequently mentioned limitations involving activities such as using sauna (65.0 %), engaging in sports (78.6 %), social interactions involving alcohol consumption (22.3 %), and eating spicy food (14.6 %).

Regarding the primary goals of laser treatment, the majority of patients (82.0 %) stated a reduction in telangiectasia, followed by 70.1 % who sought improvement in persistent erythema. Additionally, 50.1 % wished to address transient erythema, while a smaller proportion of patients sought treatment of rosacea‐associated edema (20.8 %) and burning sensations (20.2 %).

#### Tolerability, safety, and satisfaction with treatment

In terms of treatment tolerability and safety, the severity of side effects, including swelling, redness, and crusting, was rated at an average of 3.1 ± 1.4 (1–6) on a six‐point scale. The reported downtime following laser treatment varied significantly among patients, with a mean duration of 1.6 ± 3.3 days (0 – 22.5). Despite these side effects, laser therapy was generally well tolerated, with patients rating its tolerability at 4.5 ± 1.4 (1–6). Satisfaction with treatment outcomes was also high, with an average score of 4.1 ± 1.8 (1–6). A summary of the questionnaire results is provided in the online supplementary Material S3.

### Delphi consensus

#### Panelists

The necessity for a national consensus guideline on laser and energy‐based treatments for rosacea was first recognized in 2022 during the annual meeting of the *German Dermatological Laser Society* (Deutsche Dermatologische Lasergesellschaft, DDL). Leading society members and experts in rosacea management were appointed to develop evidence‐based recommendations for laser and energy‐based modalities in rosacea treatment. Of 23 invited experts, 16 physicians agreed to participate (7 did not respond). All were board‐certified dermatologists. Over a 16‐month period of interactive scientific exchange and voting, a consensus was established.

#### Delphi rounds and consensus process

**FIGURE 1:**
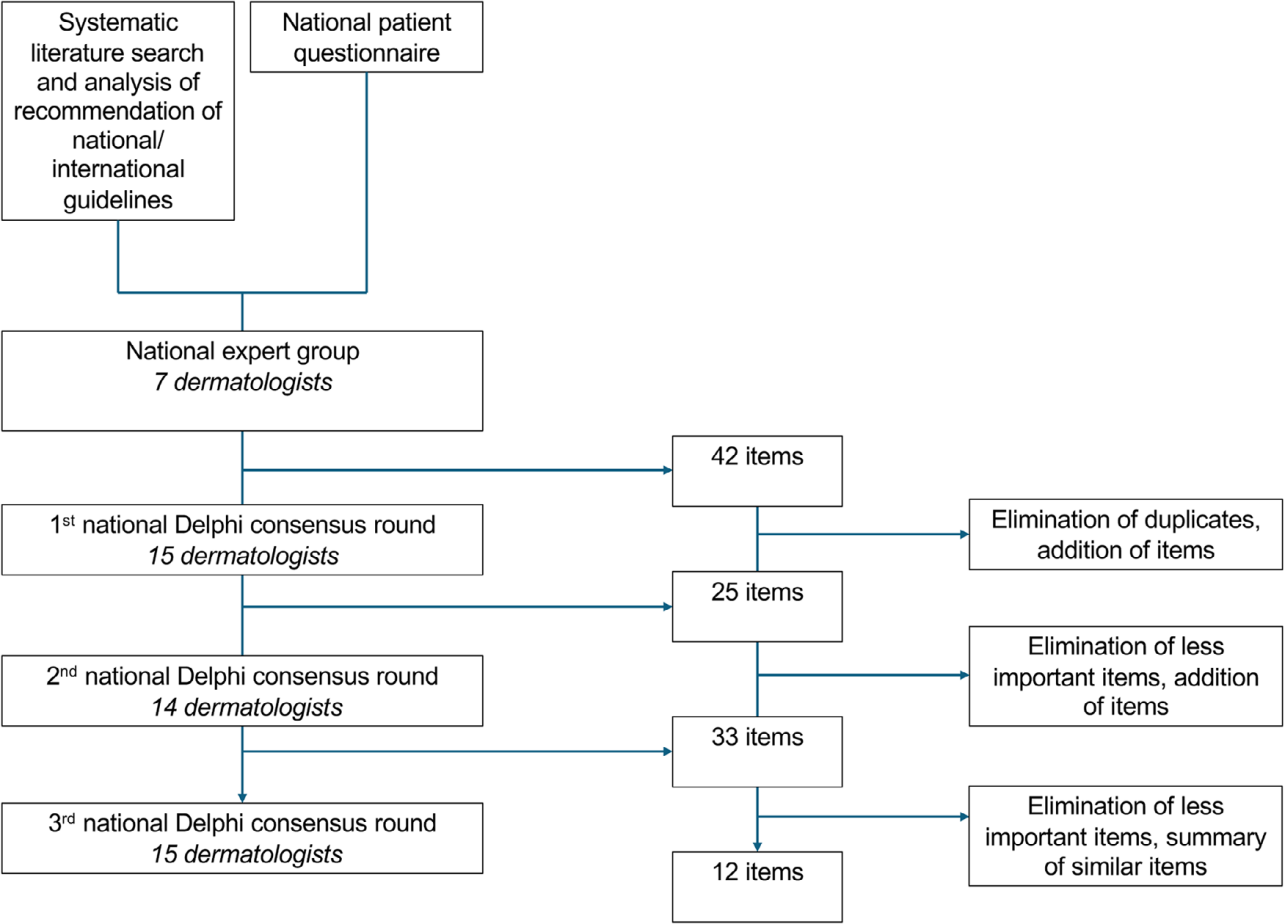
Workflow of developing the consensus statements on the use of laser and energy‐based devices in the treatment of rosacea.

The consensus process was conducted in three sequential rounds. The first round included 25 statements, the second round comprised 33 statements, and the third round addressed 12 statements. The process began with the first round in May 2023, followed by the second round in August 2023, and the final round in August 2024 (Figure 1). The Delphi statements have been categorized into three sections: Table 1 presents recommendations on laser and light‐based therapies for different rosacea phenotypes, Table 2 outlines guidance on combination therapies, and Table 3 provides recommendations for pre‐ and post‐treatment care related to laser and energy‐based procedures for rosacea.

**TABLE 1:** Consensus statements on laser and light‐based treatments for different phenotypes in rosacea.

No.	Statement	Strength	Agreement
1	Laser and light‐based treatments can be recommended for the symptomatic management of transient centrofacial erythema associated with rosacea. The erythema should be inducible during the treatment session.	**↑↑**	84.6%
2	Laser and light‐based treatments are recommended for the symptomatic management of persistent centrofacial erythema in rosacea.	**↑↑**	92.3%
3	Laser and light‐based treatments are recommended for the symptomatic management of telangiectasia in rosacea.	**↑↑**	100%
4	Laser and light‐based treatments can be recommended as a complementary treatment option for the symptomatic management of the inflammatory component of rosacea.	**↑**	92.3%

**TABLE 2:** Consensus statements on combination therapies with laser and light‐based treatments for phenotypes in rosacea.

No.	Statement	Strength	Agreement
1	For severe or refractory cases of transient centrofacial erythema in rosacea, a combination of topical brimonidine, topical metronidazole, or topical ivermectin with laser and light‐based treatments can be recommended.	**↑**	100%
2	For severe or refractory cases of persistent erythema in rosacea, a combination of laser and light‐based treatments with topical brimonidine, topical metronidazole, topical azelaic acid, or topical ivermectin is recommended.	**↑↑**	100%
3	In severe or refractory cases of the inflammatory component of rosacea, laser and light‐based treatments can be recommended as an adjunct to combination treatment with a topical agent (ivermectin, metronidazole, or azelaic acid) and a systemic agent (doxycycline or isotretinoin).	**↑**	76.9%
4	For the treatment of phymata, laser and light‐based treatments are recommended. Additionally, low‐dose systemic isotretinoin or doxycycline, or topical metronidazole, topical azelaic acid, or topical ivermectin can also be recommended as part of the treatment approach.	**↑↑**	84.6%

**TABLE 3:** Consensus statements on pre‐ and post‐care for laser and light‐based treatments for rosacea.

No.	Statement	Strength	Agreement
1	Before and after laser and light‐based treatments, protecting the affected areas from UV radiation is recommended.	**↑↑**	100%
2	It can be recommended to pause the use of topical retinoids and topical azelaic acid before and after undergoing laser or light‐based treatments.	**↑**	76.9%
3	Systemic retinoids are not contraindications for laser and light‐based treatments in rosacea.	**↑↑**	100%

## DISCUSSION

The primary objective of this modified Delphi consensus was to assess and define the role of lasers and EBDs in the treatment of rosacea. Through a structured and systematic approach, this consensus aimed to establish evidence‐based and practical recommendations for integrating these modalities into clinical management. In addition to evaluating their effectiveness for different rosacea phenotypes, the consensus explored their use in combination therapies and outlined considerations for pre‐ and post‐treatment care.

This study builds upon the most recent German guideline for rosacea, which currently classifies lasers and EBDs as “alternative treatment options”.^4^ While this classification acknowledges their potential therapeutic role, it also underscores the need for a clearer definition of their effectiveness, safety, and place in clinical practice. By addressing this gap, the present consensus provides a more structured framework for their utilization in rosacea management.

To gain insight into the patient perspective, an online‐based survey was conducted to assess the psychosocial burden of rosacea and the experiences of patients who have undergone laser and EBD treatments in Germany. The findings reaffirmed the significant impact of rosacea on quality of life, with many patients experiencing stigmatization and social limitations due to their condition. On average, patients received approximately five treatment sessions, and more than half reported that their treatment was still ongoing. While moderate side effects such as erythema, swelling, and crusting were commonly reported, these reactions were transient, typically resolving within a few days. Notably, participants expressed high levels of satisfaction with their treatment outcomes, reinforcing the efficacy of lasers and EBDs in addressing rosacea‐related concerns.

Existing literature and expert opinions support the use of laser and EBD treatments for managing persistent centrofacial erythema and telangiectasia.^4^, ^12^ Additionally, while transient erythema has been less frequently studied, expert consensus suggests that it may also be improved through targeted laser and EBD interventions, particularly when the erythema can be provoked during treatment. These technologies rely on the principle of selective photothermolysis, allowing precise targeting of dilated blood vessels while minimizing damage to surrounding tissues.^13^

For inflammatory lesions associated with rosacea, laser and EBD treatments may offer additional benefits when used in conjunction with topical or systemic pharmaceutical therapies. Combination approaches have been widely recommended for severe and treatment‐resistant cases, with physical treatment modalities such as lasers and EBDs serving as valuable adjuncts.^4^ However, further research is needed to determine the effective combinations and treatment protocols for different rosacea phenotypes.

Another critical aspect of laser and EBD treatment in rosacea is proper pre‐ and post‐care. One of the most important considerations is sun protection, as exposure to UV radiation can increase the risk of post‐inflammatory hyperpigmentation, hypopigmentation, and burns. Patients undergoing laser or EBD treatments must be thoroughly educated on the importance of photoprotection to optimize results and minimize adverse effects.

A particularly debated topic is the use of lasers and EBDs in patients receiving systemic isotretinoin therapy. Isotretinoin was long considered a contraindication for laser procedures due to concerns about impaired wound healing and an increased risk of scarring or keloid formation. This assumption was primarily based on reports from the 1980s and 1990s, which documented two isolated cases of keloid formation following argon and pulsed‐dye laser treatments.^14^, ^15^ However, more recent evidence suggests that isotretinoin should no longer be considered an absolute contraindication for laser and EBD treatments.^16^ Provided that appropriate safety protocols are followed, these procedures can be safely performed in patients on isotretinoin therapy. Based on expert consensus, it is recommended to temporarily pause the use of topical isotretinoin and azelaic acid before and after laser or EBD treatment to minimize potential irritation and adverse skin reactions.

This study has some limitations. The majority of participants in the consensus group were laser dermatologists with specialized expertise in this field, which may introduce a bias. To ensure a more balanced perspective, we included other rosacea experts in the panel. Nevertheless, future studies should aim for broader interdisciplinary representation to further enhance the comprehensiveness of these recommendations. Furthermore, the present consensus focused exclusively on the laser and energy‐based devices currently included in the guidelines. Future research should extend these discussions to emerging therapeutic modalities, such as cold atmospheric plasma.

This consensus serves as a guidance tool for clinicians managing rosacea, particularly for integrating laser and EBD treatments into individualized treatment plans. In conclusion, this Delphi study addresses key aspects of laser and EBD treatments for rosacea that required expert consensus due to recent advances in research and technology. The findings reinforce the importance of individualized treatment strategies and highlight the role of these modalities in improving patient outcomes. By providing a structured, evidence‐based framework, this study supports informed discussions between physicians and patients regarding the efficacy, safety, and practical considerations of laser and EBD treatments in rosacea management.

## FUNDING

This study was supported by the *German Society for Dermatological Laser Medicine* (Deutsche Dermatologische Lasergesellschaft, DDL).

## CONFLICT OF INTEREST STATEMENT

L.N. has received lecture fees from Cynosure Lutronic. U.P. reported research support and loan of devices from GME, Alma Lasers, Fotona, Zimmer, ThermoCeuticals, Landsberg First Class Aesthetic, and royalties from Quintessenz Publishing. C.B. receives lecture fees from L'Oréal, Asclepion Lasertechnologies, Cutera, Pierre Fabre, and P&M Cosmetics. K.F. reported receiving lecture fees from Cutera, Cynosure Lutronic, Palomar, and Juzo. K.H. has received lecture fees from Cynosure Lutronic. The other authors declare no conflict of interest.

## Supporting information



Supplementary information

Supplementary information
