# Supplementary material for: National Delphi consensus on laser and energy‐based treatments for rosacea
Source: J Dtsch Dermatol Ges. 2025 Dec 19;24(8):1070–6. doi: 10.1111/ddg.15976 (PMC13434986; doi:10.1111/ddg.15976)
Supplement: Supplementary file 2 [file DDG-24-1070-s002.docx]

| **Domain** | **Parameter** | **Value / Statistic** |
| --- | --- | --- |
| **Basic Characteristics**  **Supplementary Material S3** | Number of participants | 185 |
|  | Age (mean ± SD, minimum – maximum) | 53.5 ± 11.7 years (27 – 81) |
|  | Male/ female (%) | 21.3/ 78.7 |
|  | Average number of laser sessions (mean ± SD, minimum – maximum) | 5.1 ± 5.5 (1 – 40) |
|  | Patients completed treatment (%) | 42.5 % |
|  | Number of sessions in completed treatment (mean ± SD, minimum – maximum) | 3.3 ± 2.8 (1 – 12) |
| **Psychosocial Impact** | Negative effect on daily life  scale from 1 (none) – 6 (severe) | 3.9 ± 1.5 (1-6) |
|  | Perceived stigmatization  scale from 1 (none) – 6 (severe) | 2.4 ± 1.5 (1-6) |
|  | Restrictions in daily life (%) | 64.4 |
|  | Most affected activities (%) | Sauna: 65.0; Sports: 78.6; Social drinking: 22.3; Spicy food: 14.6 |
| **Primary Goals of Laser Treatment** | Reduce telangiectasia (%) | 82.0 |
|  | Improve persistent erythema (%) | 70.1 |
|  | Improve transient erythema (%) | 50.1 |
|  | Reduce rosacea-associated edema (%) | 20.8 |
|  | Reduce burning sensations (%) | 20.2 |
| **Tolerability, Safety, patient satisfaction** | Severity of side effects  scale from 1 (none) – 6 (severe) | 3.1 ± 1.4 (1-6) |
|  | Downtime after treatment (mean ± SD, minimum – maximum) | 1.6 ± 3.3 days (0-22.5) |
|  | Tolerability of treatment  scale from 1 (not at all) – 6 (very) | 4.5 ± 1.4 (1-6) |
|  | Satisfaction with treatment outcomes  scale from 1 (not at all) – 6 (very) | 4.1 ± 1.8 (1-6) |

**Supplementary Material S3: Summary of Patient Questionnaire Results on Experiences with Laser and Energy-Based Devices for Rosacea Treatment**
